# Philadelphia Medical Society

**Published:** 1893-11

**Authors:** 


### PHILADELPHIA MEDICAL SOCIETY.

We are glad to see prominent societies of medicine endorsing and commending the bravery of our friend, Dr. Reeves, in his successful fight against the "Amick's Cure" people.

Dr. Reeves deserves the endorsoment of every member of the profession for his course, and we are glad to join in giving to him our heartiest words of praise.

The following resolutions were recently presented to the Philadelphia County Medical Society by Dr. S. Solis-Cohen and adopted:

"*Whereas*, Dr. James E. Reeves, of Chattanooga, Tenn., having denounced the so-called "Amick Cure" for Consumption as a quack nostrum, and stated that its proprietor was not a physician in good and regular standing, was accused of criminal libel; and

"*Whereas,* the Grand Jury has ignored the indictment brought against him, be it

"*Resolved* That the Philadelphia County Medical Society congratulates Dr. Reeves on his bravery, a bravery unfortunately too rare at the present day, and tenders him sympathy in the persecution to which he has been subjected.

"*Resolved,* That no person who makes, deals in or advertises as a cure a quack nostrum, that is to say a preparation the composition of which is kept secret, can be termed a physician in good and regular standing, because such action is *ipso facto* sufficient to cause forfeiture of membership in this or any other county medical society governed by the laws of the American Medical Association.

*Resolved,* That a copy of these resolutions, duly attested with the signatures of the President and Secretary and with the seal of the society, be forwarded to Dr. Reeves, and that they be handed to the press for publication."

